# ScanGrow: Deep Learning-Based Live Tracking of Bacterial Growth in Broth

**DOI:** 10.3389/fmicb.2022.900596

**Published:** 2022-07-19

**Authors:** Ross Michael Worth, Laura Espina

**Affiliations:** ^1^Riverwell Consultancy Services Ltd., Cardiff, United Kingdom; ^2^Ineos Oxford Institute for Antimicrobial Research, Department of Zoology, University of Oxford, Oxford, United Kingdom

**Keywords:** bacterial growth, growth curves, scanner, machine learning, image classification

## Abstract

Monitoring the growth of bacterial cultures is one of the most common techniques in microbiology. This is usually achieved by using expensive and bulky spectrophotometric plate readers which periodically measure the optical density of bacterial cultures during the incubation period. In this study, we present a completely novel way of obtaining bacterial growth curves based on the classification of scanned images of cultures rather than using spectrophotometric measurements. We trained a deep learning model with images of bacterial broths contained in microplates, and we integrated it into a custom-made software application that triggers a flatbed scanner to timely capture images, automatically processes the images, and represents all growth curves. The developed tool, ScanGrow, is presented as a low-cost and high-throughput alternative to plate readers, and it only requires a computer connected to a flatbed scanner and equipped with our open-source ScanGrow application. In addition, this application also assists in the pre-processing of data to create and evaluate new models, having the potential to facilitate many routine microbiological techniques.

## Introduction

Turbidimetric measurements of bacterial cell suspensions have been, for many decades, the standard methodology to determine the concentration of bacterial cell suspensions. UV–visible spectrophotometers are commonly used for this purpose. In these instruments, samples are subjected to a light source of around 600 nm which, after passing through the suspension, is detected by a sensor and recorded as optical density (OD_600_). Bacterial cells in suspension scatter the light so the higher the cell density, and therefore more turbid the sample, the less amount of light will reach the detector, giving higher OD_600_ values (Koch, 1970). Spectrophotometers use the absorbance detection mode to convert this attenuated transmittance of light in OD_600_ units; however, only pigmented bacteria cause significant light absorption in addition to scattering, so “OD_600_” is a more accurate term than “absorbance” in microbiology.

In adequate culture broth and environmental conditions, bacterial cells in a sample replicate and the OD_600_ values evolve over time in characteristic patterns known as growth curves. A typical growth curve shows four distinct growth stages (lag phase, exponential phase, stationary phase, and death phase), each corresponding to different physiological states in the population (Zwietering et al., 1990). Live tracking of bacterial growth curves by OD_600_ measurements is one of the most common techniques in microbiology laboratories (Ram et al., 2019; Yallapragada et al., 2019). Among other applications, it is used to characterize bacterial physiology and metabolism, perform microbial fitness determinations, prepare bacterial samples at optimal growth states, monitor biomass accumulation, and study antibiotic susceptibility (Ram et al., 2019; Yallapragada et al., 2019). Currently, antibiotic susceptibility testing (AST) is especially relevant given the dramatic emergence of multi-drug-resistant bacteria. The gold standard for AST is the broth microdilution assay. In this test, initially, clear bacterial suspensions are incubated in the presence of different antibiotic concentrations, which are considered inhibitory if the turbidity of the sample does not increase after the incubation period (Andrews, 2001). Furthermore, monitoring of the growth curves during AST provides additional data that can be useful, for example, to study bacterial resistance mechanisms (Li et al., 2016) or to detect weak antimicrobial activity during screening processes.

Live tracking of bacterial growth is commonly performed in multi-well microplates to maximize sample throughput. These microplates are read by table-top absorbance plate readers or special spectrophotometers, which are expensive bulky machines limited to run a single plate (containing a limited number of samples). In recent years, cheap DIY alternatives to plate readers have arisen, based on the assembly of electronic components (LEDs, photodiodes, and Arduino microcontroller; Jensen et al., 2015; Kutschera and Lamb, 2018; Sasidharan et al., 2018), or a fitness tracker (Yallapragada et al., 2019). Laser speckle imaging has also proven effective to monitor bacterial growth (Loutfi et al., 2020). Only one of these useful devices (Jensen et al., 2015) could be applied in high-throughput settings, and all of them require advanced skills and/or components in order to function. Another ingenious method, ScanLag (Levin-Reisman et al., 2010), was designed to automatically trigger commercial flatbed scanners to acquire images of agar plates containing microbial colonies and consequently monitor the increasing size of the colonies. Inspired by this method, we aimed to design a software tool that employs flatbed scanners to acquire images of bacterial cultures in microplate wells. Like the light source in a spectrophotometer, the LED-type light source from flatbed scanners gets more scattered the more turbid the sample is, creating different patterns of detected images according to the bacterial density. In this aspect, classification of images based on their morphometric features is currently greatly facilitated by the branch of artificial intelligence known as deep learning (DL), which is a subcategory of machine learning (Sommer and Gerlich, 2013). More specifically, image classification performed by deep neural networks (the foundation of deep learning) can infer the rules to discriminate between predefined classes of exemplary images, and automatically use those rules to train a classification model. Successful application of machine learning- and DL-based image classification has been achieved for many purposes in cell biology (Sommer and Gerlich, 2013) and to specific microbiological applications (Goodswen et al., 2021). However, to the extent of our knowledge, no previous work has utilized DL to classify scanned images of bacterial cultures.

The objectives of this work were (i) to explore a new low-cost and high-throughput way of monitoring bacterial growth through the DL-enabled classification of microplate images acquired with conventional flatbed scanners and (ii) demonstrate the feasibility of this principle by developing a proof-of-concept software tool.

## Materials and Methods

### Data Acquisition

Two types of data were obtained in the data acquisition process for the training and evaluation of the DL model: scanned images of 96-well microplates containing bacterial cultures, and MS Excel files containing optical density (OD) values of the cultures in the microplates. Cultures of *Escherichia coli* MG1655 were prepared by inoculation of single colonies from agar plates into sterile universal tubes with LB (Lysogeny Broth) broth, and subsequent incubation overnight at 37°C and 120 rpm. Grown cultures were diluted into fresh LB broth to achieve different initial concentrations (ranging from 10^4^ to 10^9^ CFU/ml). 100 μl of these initial cultures was inoculated onto wells of clear non-treated flat-bottom microplates, either non-sterile (Nunc 96 MicroWell 96-Well, Thermo Scientific, Waltham, MA, United States) or sterile (Corning 96 Well, NY, United States) depending on whether subsequent incubation was performed. This type of microplate contains a maximum of 96 different cultures in 96 wells. For each training or evaluation image, a microplate was scanned in the dark (to avoid any effect of stray light) using a CanoScan LiDE 220 (Canon, Tokyo, Japan), and the optical density (OD) values of the cultures were measured at 620 nm on a microplate reader (EZ Read 400, Biochrom, Cambridge, United Kingdom, and Multiskan FC, Thermo Fisher Scientific, Waltham, MA, United States). OD_620_ values were taken without blank subtraction and stored in spreadsheet format. A total of 30 scanned images of microplates (containing 2,880 well images) were used for the training of the DL model and nine additional images were added for its evaluation.

For the Sample run testing the practicality of use of ScanGrow, a microplate was prepared by inoculating wells with *E. coli* MG1655 culture at an initial concentration of 10^4^ CFU/ml and with added antibiotics in selected wells. Negative controls with only broth were also included. The microplate was placed on the scanner, and the scanner was placed in the dark inside an incubation chamber at 37°C (S.I. 600, Stuart Scientific, Stone, United Kingdom). The scanner was triggered by the ScanGrow application to acquire images every 30 min for 46 h.

Another run to test ScanGrow in different atmospheric conditions was performed by connecting the scanner to a Raspberry Pi 3 model B+ (Sony UK TEC, Pencoed, United Kingdom), and introducing both of them in an anaerobic chamber (DG250, Don Whitley Scientific, Bingley, United Kingdom). The instructions for this special configuration are included in the quick start guide.

High-quality images were also taken for comparison purposes using another flatbed scanner (Epson Perfection V800 Photo, Suwa, Japan) and OD_600_ values were measured using a microplate reader (Synergy H1, BioTek, Vermont, United States).

### Software Application Development

ScanGrow was developed in the C# language using Microsoft.NET core 3.1 framework. The application was structured in two projects: the first a project providing the image classification features and the second the graphic user interface (GUI) and the image processing functions.

#### Provision of Image Classification Features *via* Deep Learning

**Figure 1:**
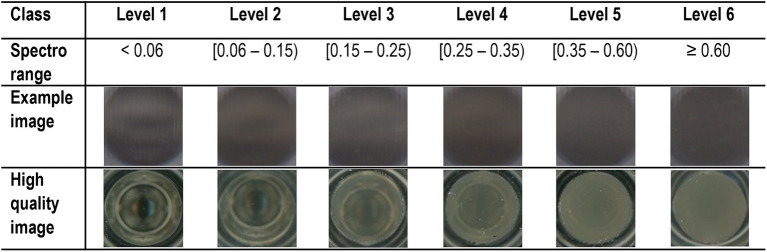
Classification of the scanned images in six classes or levels. Levels were assigned according to the corresponding intervals of spectrophotometric (OD_620_) values. Each level is represented by an exemplary image of a microplate well containing a culture of *Escherichia coli* at that specific density stage. All the images used within the ScanGrow application were acquired with a single scanner and presented the same resolution as the upper set of pictures. An additional second set of high-quality images (acquired with a high-end scanner) illustrates how these microplates look under the naked eye.

This task was achieved by integrating a DL image classification model into the ScanGrow workflow. For this, the previously obtained training dataset (consisting of raw scanned images and spreadsheets with spectrophotometric data) had to be processed into sample well images divided into classification levels. A workflow to assist with this pre-processing step was included in the ScanGrow application, and is explained in section “Additional Functions”. For the purposes of this paper, we used six classification levels, representing increasing bacterial population densities. These were decided after visual inspection of the training images and were established as follows: level 1: <0.06, level 2: [0.06–0.15), level 3: [0.15–0.25), level 4: [0.25–0.35), level 5: [0.35–0.60), and level 6: ≥ 0.60. This information is stored in a CSV file (“Classification Levels”) and imported into the application using CsvHelper (Close, 2009). An exemplary image for each classification or density level can be seen in Figure 1 (upper set of images). The CSV file gets installed in the Program Files folder and can be easily modified for the customization of the density levels.

For the proof-of-concept version of ScanGrow, the 30 images of the training dataset generated 1,196, 193, 144, 167, 674, and 506 well images corresponding to levels 1, 2, 3, 4, 5, and 6, respectively. The DL model was created by feeding these classified well images into Microsoft’s ML.NET Model Builder component in Visual Studio 2019 (v16.9.0, Microsoft, Redmond, WA, United States). ML.NET is an open-source GUI model training utility that results in a TensorFlow-compatible model. ML.NET automatically selected a CNN (Convolutional Neural Network) with ResNet-50 architecture as the highest performing model. CNNs are the most prominent class of deep neural networks and are most commonly applied to analyze visual imagery (Goodswen et al., 2021). They are comprised of nodes organized into layers following different architectures (Pak and Kim, 2017; Goodswen et al., 2021), one of which is the ResNet (Residual Networks) with 50 layers. The resulting classification model was then exported as a ZIP file and integrated into the ScanGrow application as a.dll assembly. Our software was written to easily consume any other TensorFlow-compatible classification model located in the Program Files folder.

#### GUI and Basic Image Processing Functions

**Figure 2:**
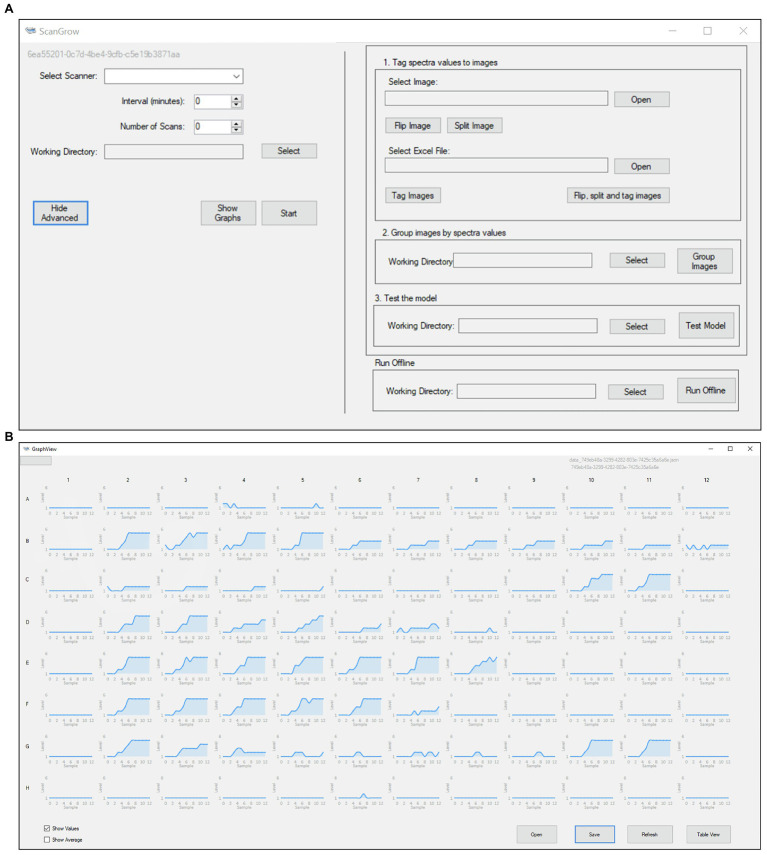
Graphic User Interface of the ScanGrow application: **(A)** Main application menu, which grants access to all the functionalities from a single window; **(B)** Graph View, which allows for the visualization of 96 different growth curves.

ScanGrow was implemented as a standalone application with a straightforward GUI. The main window of the GUI is shown in Figure 2A. The button “Start” was configured to perform an online run after the selection of a scanner device, leading to the automatic completion of a series of tasks: (i) to trigger the scanner to capture the configured number of images separated by the specified interval, (ii) to pre-process the scanned images, (iii) to assign a density level to each bacterial culture, and (iv) to sequentially store datapoints. These functions are briefly described below.

For scanner functionality, the DNTScanner. Core package (Nasiri, 2019) was implemented. ScanGrow recognizes wired or wireless scanners using the protocol WIA (Windows Image Acquisition) and triggers the scanner to acquire the specified number of images at each specified time. A4-sized images are captured at a maximum of 300 dpi (dots per inch) or the highest available resolution below this limit.

Prior to image classification, the workflow includes a pre-processing stage which flips and crops each image into 96 slices (one per microplate well). The co-ordinates and labels for each slice of the scanned image are stored in an image mask (CSV file), which are imported using CsvHelper. Each slice is automatically named according to the scanning order and stored in its corresponding folder according to the position of the well. Like with the density levels, the image mask can also be easily customized to adjust for the position of the microplate or the size of the wells.

ScanGrow loads the image classification model to predict the density level of each well at each specified time. The results of each session are saved in a JSON (JavaScript Object Notation) file and are displayed either in graphic format (Figure 2B, Graph View) or as a table grid (Supplementary Figure 1, Table View). LiveCharts (Rodriguez, 2017) was used for graph construction. We included two options to export results. First, data displayed on the Table View can be copied and pasted onto MS Excel or other spreadsheet managers; second, JSON files can be transferred between machines and loaded into ScanGrow to view results from previous sessions.

#### Additional Functions

Although initially only the online run was in our roadmap, other functions were needed along with the design and development stage and were therefore incorporated into the ScanGrow application. The option to obtain growth curves from previously acquired images became indispensable, so a job to perform offline runs (triggered by the button “Run Offline,” Figure 2A) was created. Scangrow was also supplied with functions to assist with the training and evaluation of new image classification models. The workflow to process raw training data into adequately grouped well images contains the following steps: (i) import of spectrophotometric data (in MS Excel or CSV format), (ii) import, flipping and slicing of a scanned image, (iii) automatic renaming of the images according to its spectrophotometric value, and (iv) sorting of the slices into the folder that represents their classification level. These tasks can be launched using the menu sections “1. Tag spectra values to images” and “2. Group images by spectra values” (Figure 2A). Finally, the function shown as “3. Test the model” compares the OD values provided externally (i.e., by using a spectrophotometer) with those assigned by ScanGrow. We used this function to obtain a CSV file from which the accuracy and error metrics of the classification model can be calculated.

### Data Analysis, Figures, and Images

**Figure 3:**
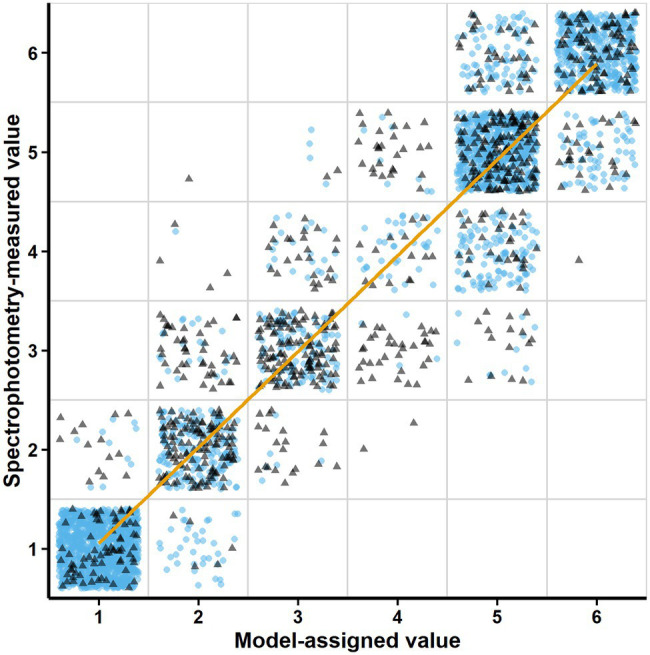
Relationship between the Model-assigned values (density levels automatically assigned to the scanned images according to the trained model) and the Spectrophotometry-measured values (density levels assigned after spectrophotometric measurements). Blue circles correspond to the validation dataset (using horizontally flipped images of the training dataset). Gray triangles correspond to the test dataset (new images and spectrophotometric measurements). The trend line represents the linear regression fit (*R*^2^ = 0.95) between the assigned and the measured values.

**Figure 4:**
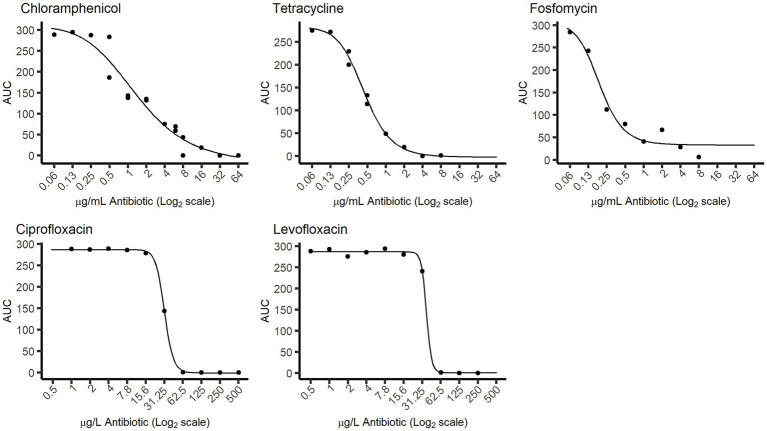
Dose–response curves obtained from the “Sample run.” The response values (points in the graph) represent the AUC values of the growth curves after 46 h of incubation, calculated directly from the 1–6 classification levels. The curves were obtained by fitting those AUC values to the dose (antibiotic concentration) values using a Hill function.

Figures 3, 4; Supplementary Figures 2, 3 were prepared by collecting raw data from the Table View in the ScanGrow application (presented as a table grid), pre-processing it in MS Excel (v2016, Microsoft), processing it in R (RStudio v1.2.1335), and using the R package ggplot2 (Wickham, 2011) for the graphic representations.

Two determinations were done for the preparation of Figure 4. First, the area under the curve (AUC) was calculated for each growth curve as a measure to quantify the general level of growth after a specific incubation time (Tonner et al., 2017). For this, the 2-point closed Newton-Cotes formula (trapezoidal rule) was applied:


$$AUC=\overset{n-1}{\underset{i=0}{\sum }}\left(\frac{f\left(t_{i}\right)+f\left(t_{i+1}\right)}{2}-1\right)$$

where *t* = each timepoint, and *f(t_i_)* = density level for each timepoint *i*, equivalent to *y_(t)_*, and taken as the density level assigned by the DL model (range 1–6). Lastly, a pharmacodynamics Hill function (Regoes et al., 2004) was adapted to express, for each antibiotic, the change in AUC as a function of the antibiotic concentration:


$$\mathrm{AU}C_{\left(x\right)}=\mathrm{AU}C_{\max}-E_{m}•\left(\frac{{\left(\frac{x}{EC_{50}}\right)}^{k}}{1+{\left(\frac{x}{EC_{50}}\right)}^{k}}\right)$$

where *x* = antibiotic concentration in log_2_ scale, *AUC_max_* = maximum AUC in the absence of antibiotics, *E_m_* = *AUC_max_* – minimum AUC at high antibiotic concentrations, *EC_50_* = antibiotic concentration at which the AUC is at half of its maximum, and *k* = Hill coefficient, which describes the sigmoidicity of the function. Only the AUC after 46 h of incubation time was needed to obtain these dose–response curves.

The image editing software Corel PhotoImpact X3 (Corel Corporation, Ottawa, Canada) was used for the preparation of Supplementary Figure 2 and for readjusting the position of the microplate in some scanned images. Bulk processing of images was done using Python scripts with the OS module and the Pillow library (Clark, 2010).

## Results

### ScanGrow Functionalities

As shown in Figure 2A, the resulting ScanGrow application offers the possibility of performing online and offline runs, as well as helping in the creation and evaluation of other models for the monitoring of growth curves and other possible types of image classifications. Online runs allow the user to monitor bacterial growth in real time through the automatic scanning and processing of images and display of updated results. Offline runs produce the same outputs as online runs but, instead of triggering a scanner, previously acquired images are used as inputs. A flowchart summarizing the steps involved in the creation of online runs, offline runs, and new models is included (Supplementary Figure 4). Detailed instructions on how to operate both types of runs, how to train and evaluate new image classification models, and other configuration options can be found in the user manual.

### Evaluation of the DL Image Classification Model

The image classification model included by default in the installation package achieved an accuracy level of 86.25% according to the self-evaluation of the ML.NET Model Builder. To further validate the adequacy of ScanGrow for monitoring bacterial growth in broth, the predictive power of the image classification model was evaluated using the “Test the model” functionality. Figure 3 shows this correlation for a total of 3,744 individual well images with a global *R*^2^ value of 0.95. These well images were obtained from 30 validation images obtained after horizontal flipping of the training images, plus nine other test images not previously used. The accuracy of the model, understood as the percentage of correct predictions, was calculated to be 81.89%. However, this number varied according to each measured density level: 96.70% for level 1 (1,274 images), 85.06% for level 2 (348 images), 64.44% for level 3 (360 images), 19.83% for level 4 (242 images), 84.63% for level 5 (885 images), and 80.16% for level 6 (635 images). More detailed results on the accuracy and error metrics of the model are presented in Supplementary Table 1.

### Practical Example of Usability

To explore a practical usage of ScanGrow, a microplate containing the same initial *E. coli* inoculum with different concentrations of several antibiotics was subjected to an online run (named here the “Sample run”). The growth of the cultures was monitored for 46 h, and the first 24 h are shown in Supplementary Figure 2. Supplementary Figure 3 shows a subset of the data centered on the antibiotic chloramphenicol: the density values assigned by the DL model are shown for each timepoint for wells containing 250, 1,000, 4,000, or 16,000 μg/L of chloramphenicol. This figure also shows the fitted growth curves using the Gompertz model. As can be seen, increasing chloramphenicol concentrations led to changes in the dynamics of growth curves, achieving longer lag times and lower maximum population density. Both changes greatly influence the area under the growth curve (AUC), which therefore can be used as a single quantitative measure of the overall growth achieved at each well. Consequently, for each antibiotic, the relationship between the AUC and the concentration of added antibiotic was calculated (shown in Figure 4). These figures demonstrate that, despite the lower resolution of ScanGrow in comparison with that of a spectrophotometer or a plate reader, the produced growth curves are accurate enough to obtain distinct dose–response curves for all the tested antibiotics.

## Discussion

### Principle and Novelty

Numerous published DIY spectrophotometers (mostly intended as instructional devices for Chemistry students) make use of the Beer–Lambert law to quantify analyte concentration from measurements of light attenuation or absorption (Hosker, 2018). Some of these devices contain a source light as well as an electronic photosensor that receives the transmitted light and transform it to voltage levels (Taha et al., 2017). Other devices project diffracted spectral images that are then captured and converted into absorbance values by analyzing pixel intensities at different wavelengths (Feng et al., 2021). In the field of microbiology, other instruments have been recently developed to assess the bacterial density of cultures (Jensen et al., 2015; Kutschera and Lamb, 2018; Sasidharan et al., 2018; Yallapragada et al., 2019; Loutfi et al., 2020). These DIY devices, as well as commercial spectrophotometers and plate readers, use electronic photosensors (typically photodiodes) to measure light transmittance and therefore measure the turbidity of bacterial cultures throughout time.

We developed a completely novel way to obtain bacterial growth curves. In contrast with the aforementioned approaches of light detection *via* photodiodes or *via* analysis of the diffracted spectral images, we based the measurement of turbidimetry on the direct analysis of scanned images of bacterial cultures. The use of a flatbed scanner was inspired by the tool ScanLag (Levin-Reisman et al., 2010), used to track the size of solid colonies in agar plates. In our case, the adequacy of flatbed scanners to capture OD data is based on the geometry of their optical system: it is such that the light that is specularly reflected from the sample does not reach the photosensors, so the scanner can be used as a physical instrument for recording the scattered light (Zheleznyak and Sidorov, 2015). In the flatbed scanner used during this investigation, a linear array of LED lights illuminates the sample while photosensors (of type LIDE, an improved version of contact image sensors) capture the image. In our setup, a black cover is placed on top of the microplate to facilitate the classification of well images according to their turbidity: the light emitted by the LED lights is transmitted through the sample and the black cover is reflected back. As shown in Figure 1, the combination of reflected lights and shadows creates an image pattern that is highly recognizable in clear samples of low bacterial density but fades in accordance with the increasing turbidity of the samples.

Another relevant novel and advantageous element in this investigation is the use of deep learning (DL) for the classification of the images into density levels (equivalent to OD_620_ measurements). We employed one of the most common DL methods, supervised classification, which is based on the definition of distinct classes by predefined representative examples (Sommer and Gerlich, 2013). For this class definition, we decided to set the number of levels to six (classes shown in Figure 1) to be able to create growth curves with enough resolution without detriment of the accuracy of the DL model. Finally, we decided the ranges of each level based on visual inspection of the images. In addition to the use of DL and its integration into the user-friendly application, we incorporated built-in features to assist in the creation of user-defined DL models with customizable class definitions. To the best of our knowledge, this study not only demonstrates a novel way of obtaining bacterial growth curves but also offers a user-friendly application for that purpose, either using a predefined or customized DL model.

### Functionality and Limitations

The idea of using microplate images to monitor bacterial growth curves originated from the need to read more than 20 microplates during overnight incubation in different atmospheric conditions. In these high-throughput screenings, we needed to spot alterations in the growth curves of a target pathogen in the possible presence of antimicrobial compounds. A single traditional plate reader was obviously insufficient for this task, and a software tool had to be developed to facilitate image capture and processing for quick identification of anomalous growth curves. ScanGrow proved absolutely fit for this purpose: as shown in Supplementary Figure 3, ScanGrow can detect signs of bacteriostatic activity in growth curves, like a prolonged lag phase and a diminished maximum population density (Li et al., 2016).

We therefore developed ScanGrow as a fit-for-purpose proof-of-concept application designed to enable laboratorial analysis, and consequently, there are some compromises in the software design. The main practical limitation of the current proof-of-concept version of ScanGrow is the low resolution of the attained growth curves, as only six differentiated density levels have been established. This abridgment deems the presented version of ScanGrow unsuitable for those uses that require high data accuracy. Despite this low resolution, the dose–response curves corresponding to the fluoroquinolones levofloxacin and ciprofloxacin showed considerable higher steepness than the curves obtained for the rest of the antibiotics (Figure 4). This observation agrees with the known notion of the efficacy of fluoroquinolones being very concentration-dependent (Drusano et al., 1998), and suggests that ScanGrow can also be used for general antibiotic resistance studies – for example, for the characterization of the evolution of antibiotic resistance of bacterial strains.

Another limitation of the current version of ScanGrow is its lower accuracy predicting intermediate density levels (levels 3 and especially level 4) in comparison with the rest (Figure 3; Supplementary Table 1). Both the resolution and the accuracy of the current version of ScanGrow can be easily strengthened by increasing the number and quality of the scanned images. It should be noted that the current version of ScanGrow has been configured to use scanned A4 size-images at a resolution of 300 dpi, since all images were acquired with an economical scanner coupled with contact image sensors. As can be inferred from Figure 1, increasing the quality of the well images could be attained by using a different flatbed scanner of higher resolution or one that uses an optical system of a charge-coupled device (Zheleznyak and Sidorov, 2015). Besides, all the acquired images presented an inherent lack of optical focus due to the skirts of the microplates elevating the bottom wells away from the scanner surface. If non-skirted flat-bottom microplates were readily available, their use would be recommended to obtain in-focus images.

The default classification model has been trained using images acquired in the same laboratory settings and using the same experimental conditions (a standard *E. coli* strain grown in autoclaved LB broth under static aerobic incubation in standard flat-bottom 96-well microplates). Even the use of different autoclaves can cause different intensities of Maillard browning in the broth, possibly leading to different image patterns. Consequently, we would recommend to train and validate a new classification model so that it is adequate for each experimental setting. Similarly, the DL feature extraction process could also benefit from consistent and optimized experimental conditions during the training process. For example, different covers and sources of light could be tested before selecting the ones that create the best image contrast, and those settings should be maintained during all subsequent work. Furthermore, optimization of the training data quality and of the feature extraction process could even lead to the training of a regression model to predict numeric values instead of classes.

### Comparison With Other Devices

ScanGrow offers a series of unique advantages in comparison with commercially available plate readers and with small DIY devices recently developed for bacterial growth monitoring. The first asset is its low price and high accessibility: in contrast to the high price of plate readers (Kutschera and Lamb, 2018; Yallapragada et al., 2019), the whole ScanGrow setup only needs an incubator, a computer and flatbed scanner(s). In contrast, although the small DIY devices (Jensen et al., 2015; Kutschera and Lamb, 2018; Sasidharan et al., 2018; Yallapragada et al., 2019) are relatively cheap and portable, all require some kind of 3d printing and/or assembly of electronic components.

Second, ScanGrow has been developed to be extremely high-throughput. With one exception (Jensen et al., 2015), the aforementioned DIY devices are not high-throughput (only allowing 1–4 samples per run), while most commercial plate readers can only read one microplate with a maximum of 96 or 384 samples per run. Although at its current development stage ScanGrow only interprets one microplate per scanner, several scanners can be simultaneously connected to the same computer and run independent application sessions. Besides, improved versions of ScanGrow could include the possibility of reading five microplates per scanner, making it possible to read up to 480 samples per scanner, 960 samples if using two scanners, etc. Processing a high number of samples is of special relevance in screening tests like sterility sampling, or to help characterize bacterial growth and/or antibiotic susceptibility in extensive strain collections *via* growth curve monitoring, as previously done (Li et al., 2016).

Finally, a relevant advantage unique to ScanGrow is its versatility. ScanGrow can function in special atmospheric conditions, like temperatures below room temperature, or introduced in anaerobic chambers and controlled by a Raspberry Pi (configuration instructions are detailed in the user manual). All the small portable DIY devices share this same advantage when compared to commercial plate readers, which need expensive added features to work under special incubation conditions. Additionally, and as a unique characteristic to ScanGrow, deep-well plates or additional attachments to the microplates could be used and configured, while they would not fit on traditional instruments or 3d-printed devices. More importantly, the option of training and integrating custom DL classification models into ScanGrow opens the possibility of obtaining growth curves at different experimental conditions, like using diverse types of containers. Furthermore, other DL models could be developed for completely different applications, such as agar dilution tests (by categorizing the size of bacterial colonies) or colorimetric assays (by categorizing color intensity or tone).

### Future Development

This study presents the proof-of-concept version of ScanGrow, but several areas of improvement have been identified and added to the ScanGrow roadmap. Some of the most crucial upgrades have already been explained: the enhancement of the resolution and accuracy of the default DL model and the increase of the throughput by integrating the analysis of several microplates acquired with the same scanner. In addition, some software modifications have also been planned, namely the integration of the DL construction into the application, the improvement of the data importation and exportation, the integration of the data with a database (rather than a flat file), the automatic detection of the position of the microplates, the incorporation of the automatic modeling of growth curves, and the possibility of automatic calculation of dose–response curves after additional data input from the user.

## Conclusion

In this work, we describe a new way of monitoring bacterial growth *via* the machine learning classification of scanned images of liquid cultures in microplates. We tested the accuracy of this tool, ScanGrow, and demonstrated one of its applicability options by analyzing the effect of several concentrations of different antibiotics on bacterial growth curves.

We offer the ScanGrow application as a low-cost, user-friendly, and versatile alternative to plate readers for tests that do not require a high level of sensitivity. We consider ScanGrow low-cost as the user only has to install our open-source application in a computer with access to a flatbed scanner. Its user-friendly interface facilitates the whole process of automatic image acquisition, data processing, results visualization and exportation, and creation and evaluation of new image classification models. Since ScanGrow is highly customizable and does not require bulky instrumentation, it can be adapted to atypical cultivation conditions and to perform other laboratory assays that can benefit from automated image-based classification.

Finally, we especially advocate the use of ScanGrow or other innovative alternatives to plate readers in research laboratories in low-resource settings.

## Data Availability Statement

The DL model training dataset, the DL model testing dataset, and the Sample run can be downloaded from Figshare at https://doi.org/10.6084/m9.figshare.16822924. The source code and the installation package can be found at https://github.com/lauraespina/ScanGrow. The installation package contains a MSI installer, a user manual and a quick start guide. The original contributions presented in the study are included in the article/Supplementary Material, further inquiries can be directed to the corresponding author.

## Author Contributions

LE: conceptualization, laboratorial methodology development, data curation, data analysis, data visualization, project administration, and writing—original draft preparation. RW: software development, software testing, writing—original draft preparation (on software methodology), and writing—review of original draft. All authors contributed to the article and approved the submitted version.

## Funding

The laboratorial work was funded by the Knowledge Transfer Partnership program from Innovate UK (project number 509858 and partnership number 10315, http://ktp.innovateuk.org) until October 2019 and by the Ineos Oxford Institute for Antimicrobial Research (https://ineosoxford.co.uk) from November 2019 onward.

## Conflict of Interest

RW was employed by Riverwell Consultancy Services Ltd.

LE declares that the research was conducted in the absence of any commercial or financial relationships that could be construed as a potential conflict of interest.

## Publisher’s Note

All claims expressed in this article are solely those of the authors and do not necessarily represent those of their affiliated organizations, or those of the publisher, the editors and the reviewers. Any product that may be evaluated in this article, or claim that may be made by its manufacturer, is not guaranteed or endorsed by the publisher.
