# Supplementary material for: ScanGrow: Deep Learning-Based Live Tracking of Bacterial Growth in Broth
Source: Front Microbiol. 2022 Jul 19;13:900596. doi: 10.3389/fmicb.2022.900596 (PMC9343779; doi:10.3389/fmicb.2022.900596)
Supplement: Supplementary file 1 [file Data_Sheet_1.pdf]

Supplementary Material for:

ScanGrow: Deep Learning-Based Live Tracking of Bacterial Growth in Broth

Ross Michael Worth and Laura Espina

Front. Microbiol. 13:900596. doi: 10.3389/fmicb.2022.900596

============= Github repository: https://github.com/lauraespina/ScanGrow =================

1 Supplementary Methods

For Supplementary Fig. 3, the growth curves were fitted to Zwietering's modified Gompertz equation (Zwietering *et al.*, 1990):

$$y_{(t)} = a \cdot exp\left\{-exp\left(\frac{\mu \cdot exp(1)}{a}\right)(\lambda - t) + 1\right\} + 1$$

where $y_{(t)}$ = density level for each timepoint t, μ = maximum specific growth rate, λ = lag time, and a = -1 + value of the upper asymptote, corresponding to the maximum population density maintained during the growth period.

2 Supplementary Tables

Supplementary Table 1. Results from the evaluation of the machine learning image classification model showing the number of total wells, number of correctly predicted wells and percentage of accuracy for each density level. The coefficient of determination (R^2) of the linear regression fit between the predicted density levels and the measured density levels is also presented for each dataset. The mean absolute error (MAE) of the model for each dataset indicates that the model is off on average by that number of density levels. Results refer to the validation dataset (30 flipped images of the training dataset with their corresponding spectrophotometric measurements), the test dataset (9 new microplates with their spectrophotometric measurements) or both combined. Images in the validation dataset were created by horizontally flipping the images used for the training of the image classification model and offsetting the position of the microplate.

	Validation + Test			Validation			Test		
Density level	No. total wells	No. correct wells	% Accuracy	No. total wells	No. correct wells	% Accuracy	No. total wells	No. correct wells	% Accuracy
1	1274	1232	96.70	1196	1159	96.91	78	73	93.59
2	348	296	85.06	193	177	91.71	155	119	76.77
3	360	232	64.44	144	109	75.69	216	123	56.94
4	242	48	19.83	167	34	20.36	75	14	18.67
5	885	749	84.63	674	594	88.13	211	155	73.46
6	635	509	80.16	506	416	82.21	129	93	72.09
All	3744	3066	81.89	2880	2489	86.42	864	577	66.78
			R^2 = 0.95			R^2 = 0.97			R^2 = 0.84
			MAE = 0.19			MAE = 0.14			MAE = 0.36

3 Supplementary Figures

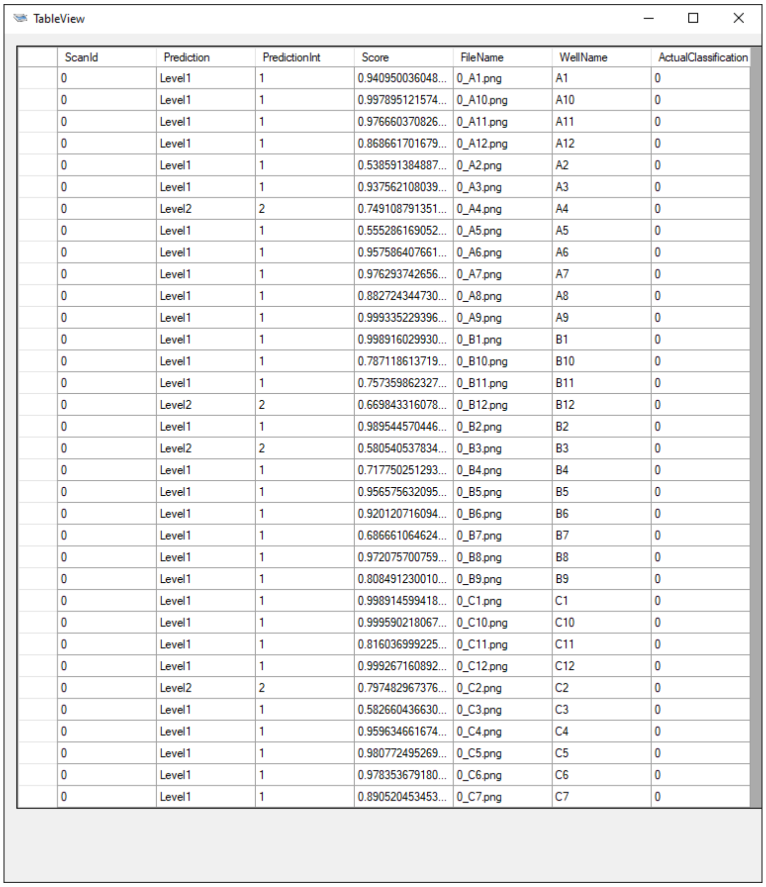

	ScanId	Prediction	PredictionInt	Score	FileName	WellName	ActualClassification
	0	Level1	1	0.940950036048...	0_A1.png	A1	0
	0	Level1	1	0.997895121574...	0_A10.png	A10	0
	0	Level1	1	0.976660370826...	0_A11.png	A11	0
	0	Level1	1	0.868661701679...	0_A12.png	A12	0
	0	Level1	1	0.538591384887...	0_A2.png	A2	0
	0	Level1	1	0.937562108039...	0_A3.png	A3	0
	0	Level2	2	0.749108791351...	0_A4.png	A4	0
	0	Level1	1	0.555286169052...	0_A5.png	A5	0
	0	Level1	1	0.957586407661...	0_A6.png	A6	0
	0	Level1	1	0.976293742656...	0_A7.png	A7	0
	0	Level1	1	0.882724344730...	0_A8.png	A8	0
	0	Level1	1	0.999335229396...	0_A9.png	A9	0
	0	Level1	1	0.998916029930...	0_B1.png	B1	0
	0	Level1	1	0.787118613719...	0_B10.png	B10	0
	0	Level1	1	0.757359862327...	0_B11.png	B11	0
	0	Level2	2	0.669843316078...	0_B12.png	B12	0
	0	Level1	1	0.989544570446...	0_B2.png	B2	0
	0	Level2	2	0.580540537834...	0_B3.png	B3	0
	0	Level1	1	0.717750251293...	0_B4.png	B4	0
	0	Level1	1	0.956575632095...	0_B5.png	B5	0
	0	Level1	1	0.920120716094...	0_B6.png	B6	0
	0	Level1	1	0.686661064624...	0_B7.png	B7	0
	0	Level1	1	0.972075700759...	0_B8.png	B8	0
	0	Level1	1	0.808491230010...	0_B9.png	B9	0
	0	Level1	1	0.998914599418...	0_C1.png	C1	0
	0	Level1	1	0.999590218067...	0_C10.png	C10	0
	0	Level1	1	0.816036999225...	0_C11.png	C11	0
	0	Level1	1	0.999267160892...	0_C12.png	C12	0
	0	Level2	2	0.797482967376...	0_C2.png	C2	0
	0	Level1	1	0.582660436630...	0_C3.png	C3	0
	0	Level1	1	0.959634661674...	0_C4.png	C4	0
	0	Level1	1	0.980772495269...	0_C5.png	C5	0
	0	Level1	1	0.978353679180...	0_C6.png	C6	0
	0	Level1	1	0.890520453453...	0_C7.png	C7	0

Supplementary Figure 1. Example of results displayed on the Table View as part of the Graphic User Interface of the ScanGrow application.

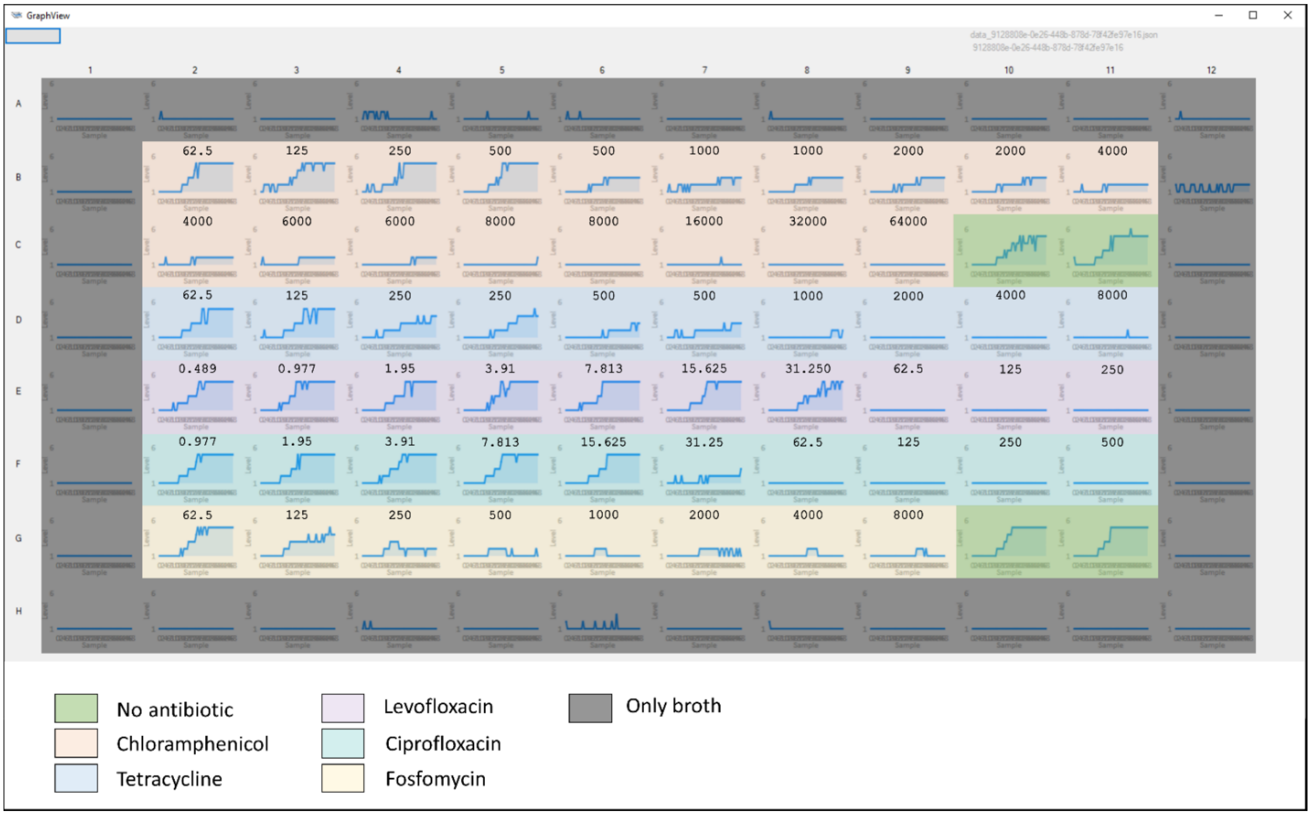

Supplementary Figure 2. Graph View showing the growth curves of *Escherichia coli* at 37 ºC (initial concentration of 10^4 CFU/mL) incorporated with different concentrations of antibiotics in the "Sample run". Negative and positive controls (only broth and only culture with no antibiotics, respectively) were also included. This graph shows the first 24 h of the run, being the complete run 46 h long with one image scanned every 30 min. Numbers written on top of the graphs represent the antibiotic concentration (in µg/L) added in each well.

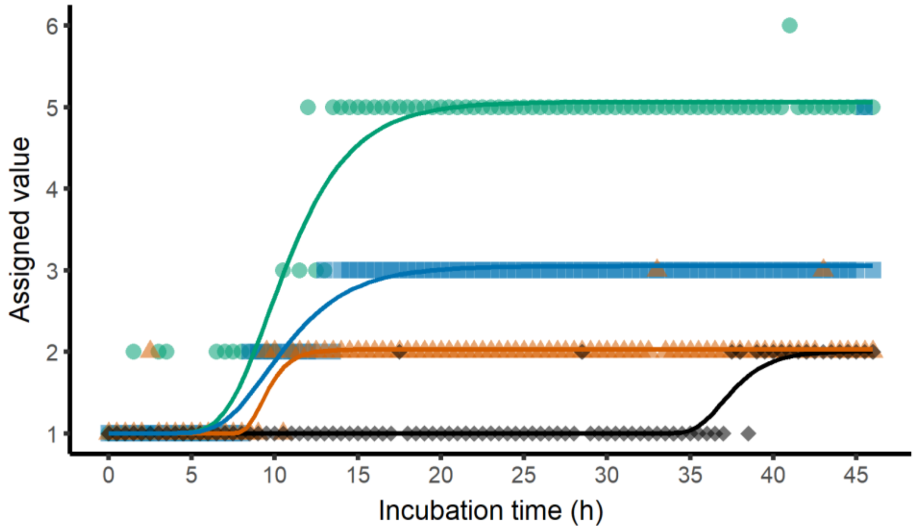

Supplementary Figure 3. Detailed growth curves from wells B4, B8, B11 and C7 in the "Sample run", corresponding to cultures added with 250 (green circles), 1000 (blue squares), 4000 (orange triangles) or 16000 (black diamond) µg/L of chloramphenicol. The data points retrieved from the Table View were fitted to the incubation timepoints (total run of 46 h) by using a Gompertz function, and the resulting fits are shown as solid lines.

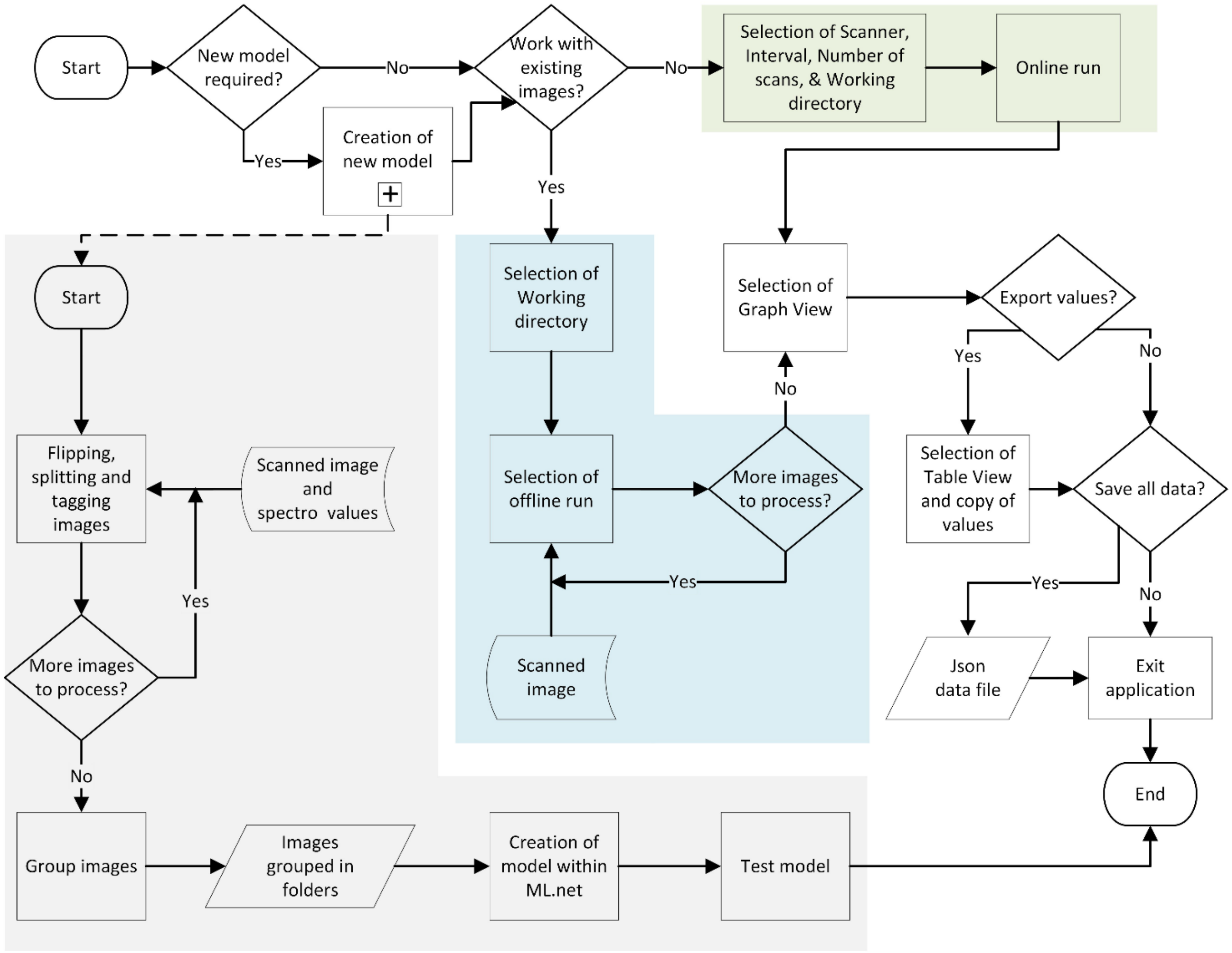

Supplementary Figure 4. Flowchart summarizing the steps involved in the creation of a new online run (shadowed in green), a new offline run (shadowed in blue) and a new classification model (shadowed in gray). Common steps to these processes are not shadowed.
